# What Happened Next? The Experiences of Postsecondary Students With Disabilities as Colleges and Universities Reconvened During the Pandemic

**DOI:** 10.3389/fpsyg.2022.872733

**Published:** 2022-05-05

**Authors:** Joseph W. Madaus, Michael N. Faggella-Luby, Lyman L. Dukes, Nicholas W. Gelbar, Shannon Langdon, Emily J. Tarconish, Ashely Taconet

**Affiliations:** ^1^Department of Educational Psychology, University of Connecticut, Mansfield, CT, United States; ^2^Alice Neeley Special Education Research and Service (ANSERS) Institute, Texas Christian University, Fort Worth, TX, United States; ^3^Exceptional Student Education Program, University of South Florida, Tampa, FL, United States; ^4^Special Education Program, University of Illinois at Urbana-Champaign, Champaign, IL, United States

**Keywords:** college students with disabilities, accessibility, instruction, postsecondary, COVID-19

## Abstract

COVID-19 caused nearly every college and university in the United States to rapidly shift to remote learning during the spring 2020 semester. While this impacted all students to different degrees, students with disabilities (SWD) faced new challenges related to their mental health, the accessibility of their instruction, the receipt of accommodations, and their interactions with faculty and student support personnel. Literature is emerging that describes the experiences of SWD during the spring 2020 semester and the swift change to remote learning. However, little is currently known about what followed for these students. The present study builds from a prior investigation of SWD during the spring 2020 semester and examines student experiences and perceptions during the 2020–2021 academic year. Eighty-eight SWD from colleges across the United States completed an instrument that contained a mix of demographic, yes/no, Likert scale and open-ended items. Responses revealed most items related to accessing services and instruction showed no improvement from the spring 2020 semester, and that items related to mental health, motivation to learn, and connections with peers were perceived as worse than in spring 2020. Open-ended responses revealed similar themes, with some students describing no improvements, and others noting that accessibility service offices and faculty provided enhanced methods of communication and support. Implications for practice and future research are presented.

## Introduction

The United States reported the largest number of COVID-19 cases in the world since the onset of the pandemic in late 2019 (World Health Organization, 2021). Predictably, colleges and universities in the United States were profoundly impacted. It is estimated that approximately 14 million college students shifted to remote learning during spring 2020, which occurred across a matter of days, due to the pandemic (Hess, 2020). The pandemic continued its significant impact into the following academic year, with more than 95% of higher education professionals reporting their institution provided a continuum of educational options including fully remote or hybrid, and in-person learning, during the fall 2020 semester (Scott and Aquino, 2021).

### Experiences of All College Students

College students in general faced a variety of academic and non-academic challenges as the pandemic unfolded. Access to a multitude of typical institutional operations were interrupted, rendering campus services either nonexistent or difficult to utilize. For example, an overarching theme reported by students was the inaccessibility of student services (e.g., financial aid and registrar offices; McDaniel et al., 2020). Academically, students reported feeling overwhelmed with the shift to remote education, especially due to a lack of consistency in the technology used to deliver their courses (McDaniel et al., 2020). In one analysis that included reports from several surveys, between 16 and 19% of students reported challenges regarding access to the necessary technology to participate in remote education (Jaggars et al., 2021).

Students also reported obstacles accessing appropriate distraction free study spaces (McDaniel et al., 2020), as well as a decreased sense of belonging, opportunities for collaboration with peers, and interest in course materials after instruction moved online (Means and Neisler, 2020; Madaus et al., 2021; Tarconish et al., in press). In another study, students reported concerns that the pandemic could jeopardize their future plans (Lederer et al., 2021). For example, Aucejo et al. (2020) stated some college students delayed graduation, with 13% of all students reporting a graduation postponement and 55% of lower-income students reporting doing so.

Numerous personal issues were also reported, ranging from housing, food, employment, and financial insecurities to an uptick in mental health diagnoses (Soria et al., 2020). In fact, a survey of more than 38,000 college students in spring 2020 determined nearly three out of five had expressed insecurity regarding basic needs as the pandemic unfolded (Goldrick-Rab et al., 2020). Specifically, 44% of two-year and 38% of four-year college students reported food insecurity, and 36% of two-year and 41% of four-year students reported housing insecurity. In the same survey, 74% of respondents indicated working prior to pandemic onset. As the country shut down, 33% of two-year and 42% of four-year students reported being laid off a job. Overall, the challenges resulted in some students questioning whether to return for the fall 2020 semester. Reported reasons that domestic students did not re-enroll at their institutes of higher education included “concerns that their classes will continue to be held online” (71%), “financial constraints” (49%), and their “experience at the university during the COVID-19 pandemic” (48%; SERU Consortium, 2020). While the pandemic presented challenges for all students, inequities in education faced by marginalized groups, including students with disabilities (SWD), were especially exacerbated by the pandemic (McDaniel et al., 2020; Aquino and Scott, 2021; Rodriguez et al., 2021) and studies are emerging that investigate the experiences of SWD specifically.

### Experiences of Students With Disabilities

Students with disabilities make up approximately 19% of all undergraduate students in the United States (United States Department of Education, 2021). Pandemic-related reports indicated postsecondary SWD faced more adversity with the shift to remote education compared to their peers without disabilities (Scott and Aquino, 2020; United States Department of Education, 2021). For example, SWD had greater concerns with remote education and indicated specific difficulty meeting novel online course requirements, resulting in lower earned grades, as well as disruptions to graduation timelines (Zhang et al., 2020). SWD also reported the negative impact of limited interaction with instructors and peers regarding course materials and activities (Madaus et al., 2021).

Although the U.S. Department of Education, Office for Civil Rights (2020) clearly advised colleges and universities that reasonable accommodations must continue to be provided to SWD regardless of the instructional delivery format, studies indicated student accommodation needs shifted, or were sometimes not fully met, during remote education when compared to in-person education (Lalor and Banerjee, 2020; Madaus et al., 2021). For example, Behling (2020) described a situation in which accessibility service staff were asked by students to remind professors that appropriate accommodations should be provided throughout the duration of remote instruction due to an increased impact on their mental health. Other SWD reported challenges providing documentation of a disability, which may be required to access accommodations (Scott and Aquino, 2020). Additionally, students who received testing accommodations such as a private room may not have been able to access those accommodations while testing at home, with some students reporting more distractions in the home environment (Valenzuela, 2020; Madaus et al., 2021).

Non-academic challenges were also present for SWD, resulting in students seeking support from accessibility services (Behling, 2020). Examples parallel those reported earlier by students regarding loss of employment, as well as job and food insecurity (Soria et al., 2020). Additionally, SWD reported issues with Wi-Fi and technology access as well as unanticipated technology expenses (Scott and Aquino, 2020; Soria et al., 2020). The pandemic also exacerbated mental health concerns. For example, SWD reported increased rates of depression, and specifically major depressive disorder that appeared to be pandemic-related (53 to 70%) as compared to peers without disabilities (34%; Soria et al., 2020; Lederer et al., 2021). In addition to mental health concerns, students reported feelings of isolation and a decreased sense of belonging on campus, which was noted more often by SWD than their peers without disabilities (United States Department of Education, 2021). Safety was another reported concern and potentially related to increased mental health challenges, as SWD were significantly more likely to live in a setting in which physical and emotional violence was present during the pandemic (25 to 41% dependent on disability) as compared to their peers without disabilities (14%; Soria et al., 2020).

Despite the many challenges encountered due to the shift to remote instruction, SWD also reported benefits. For example, students with physical disabilities or chronic health conditions were able to attend their courses without the need to commute to campus (Valenzuela, 2020; Faggella-Luby et al., 2021). Others indicated specific academic benefits to the remote environment including the ability to pay more attention to their classes (Tarconish et al., in press). Some SWD reported a reduced need to utilize certain accommodations because of increased accessibility features in remote courses such as recorded lectures, which were available through online platforms and learning management systems (LMS; Madaus et al., 2021; Tarconish et al., in press). Other benefits reported by students included being able to readily meet with peers, professors, and accessibility service professionals through web-based communication platforms (e.g., Zoom, Webex, Microsoft Teams).

### Rationale for the Present Study

While literature is emerging that explores the academic and personal experiences of SWD during the spring 2020 semester and the initial transition to remote learning, to-date there is minimal published research on the experiences of SWD in the 2020–2021 academic year. The current study addresses this void, providing a much needed and time sensitive exploration of SWDs’ educational experiences during the 2020–2021 academic year. This includes examination of student perceptions concerning what institutions and accessibility service offices did well and how their experiences in the spring 2021 semester compared to their experiences in the spring 2020 semester.

## Materials and Methods

The purpose of this mixed-methods study is to follow up on the study by Madaus et al. (2021) that focused on the experiences of SWD during the spring 2020 semester. Respondents to that study were asked if they would be interested in participating in a follow-up investigation, and 188 SWD indicated a willingness to do so and provided a contact email address. The electronic survey used in the Madaus et al. (2021) study was modified to focus on student experiences in the 2020–2021 academic year. Modifications included changing the wording of item stems related to attendance and living situation to be specific to the 2020–2021 academic year and changing the wording of item stems related to academic experiences and support receipt to be specific to the spring 2021 semester, in order to allow for longitudinal comparisons to the spring 2020 semester. In addition, the Likert-scale was changed from a Strongly Disagree to Strongly Agree scale to one that focused on the participants reflecting on the differences between Spring 2020 and Spring 2021 (i.e., Much worse than Spring 2020 to Much better than Spring 2020). Six items were added including whether a quarantine was required at any time and if the respondent tested positive for COVID-19. Affirmative answers to either resulted in follow-up questions asking about the impact of these events on the student’s academic experience. The demographic questions remained consistent across the two versions of the survey. It is important to note that participants could select multiple disability categories and non-binary gender options.

Institutional Review Board Exempt approval was received at the institution of the primary author. An invitation to participate in the study that included a link to the revised survey (available from the first author upon request) was emailed to the 188 SWD who provided an email address as part of the Madaus et al. (2021) investigation. Participants were provided a study explanation and request to participate. Student consent to participate was provided if “yes” was selected and a response of “no” resulted in those students exiting the electronic consent page. The survey was sent to potential respondents in April 2021 in three rounds approximately 12 days apart. Survey completers were offered a $25 Amazon gift card for study participation.

Of the 188 emailed students, 112 clicked the link and 111 indicated a willingness to participate in the survey. The data from the survey were downloaded into SPSS (Version 28.0) and variables for the number of missing items for the total survey, demographic questions, and Likert-scale items for each potential participant were calculated. Based on analyzing the number of missing items per person, it was found that 23 individuals only responded to the demographic questions and did not answer any content questions and were eliminated from the data set resulting in 88 viable responses for analysis. Frequencies for all items and means and standard deviations for the Likert-scale items were then calculated. We chose to focus on simple descriptive statistics so we could describe the experiences of students during the second year of the pandemic. Because our demographic questions reflected the complexity and intersectionality of these constructs, it was not possible to effectively analyze the Likert responses by disability type or gender.

### Qualitative Analysis

**Figure 1:**
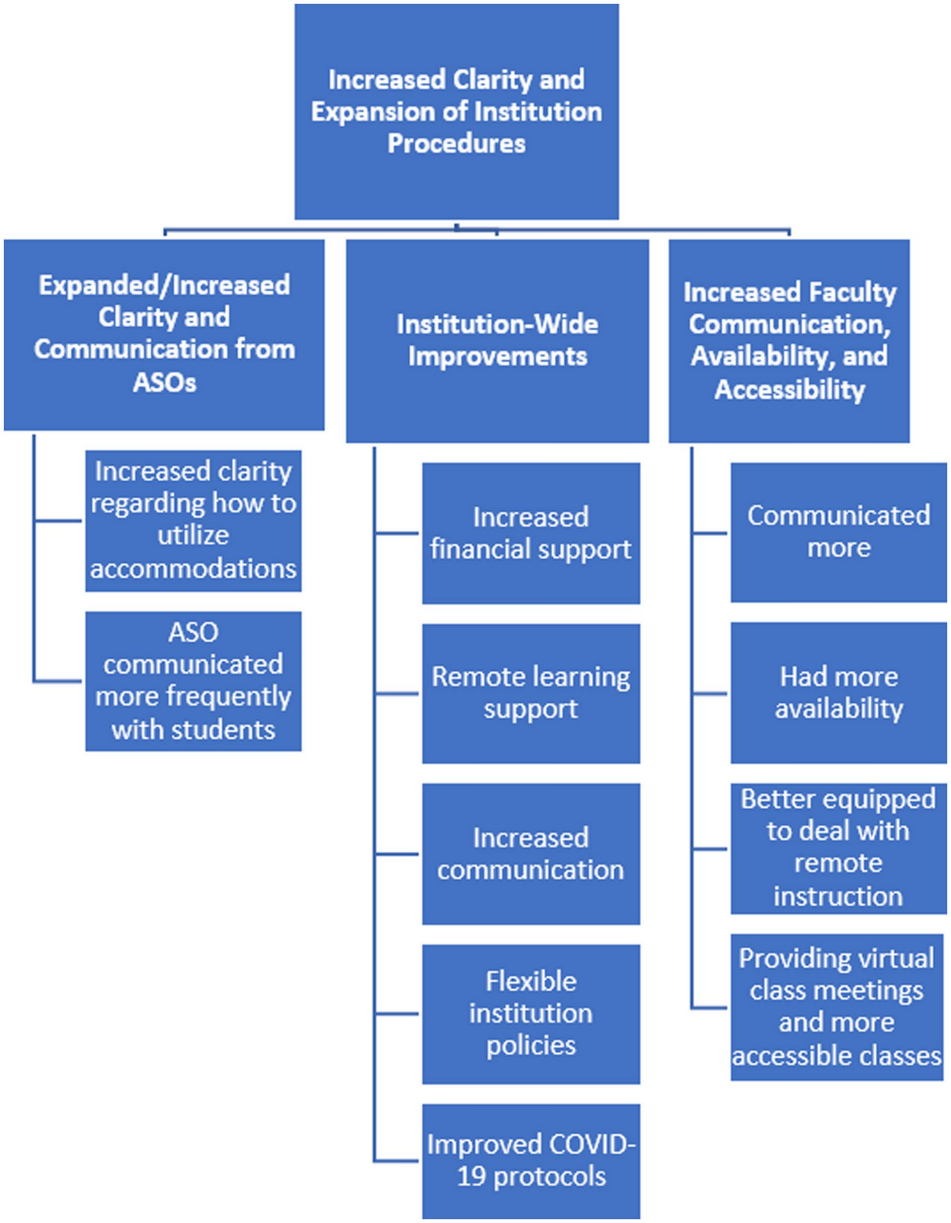
Improvements made as Institution policy and practice adapted to the pandemic coding tree.

**Figure 2:**
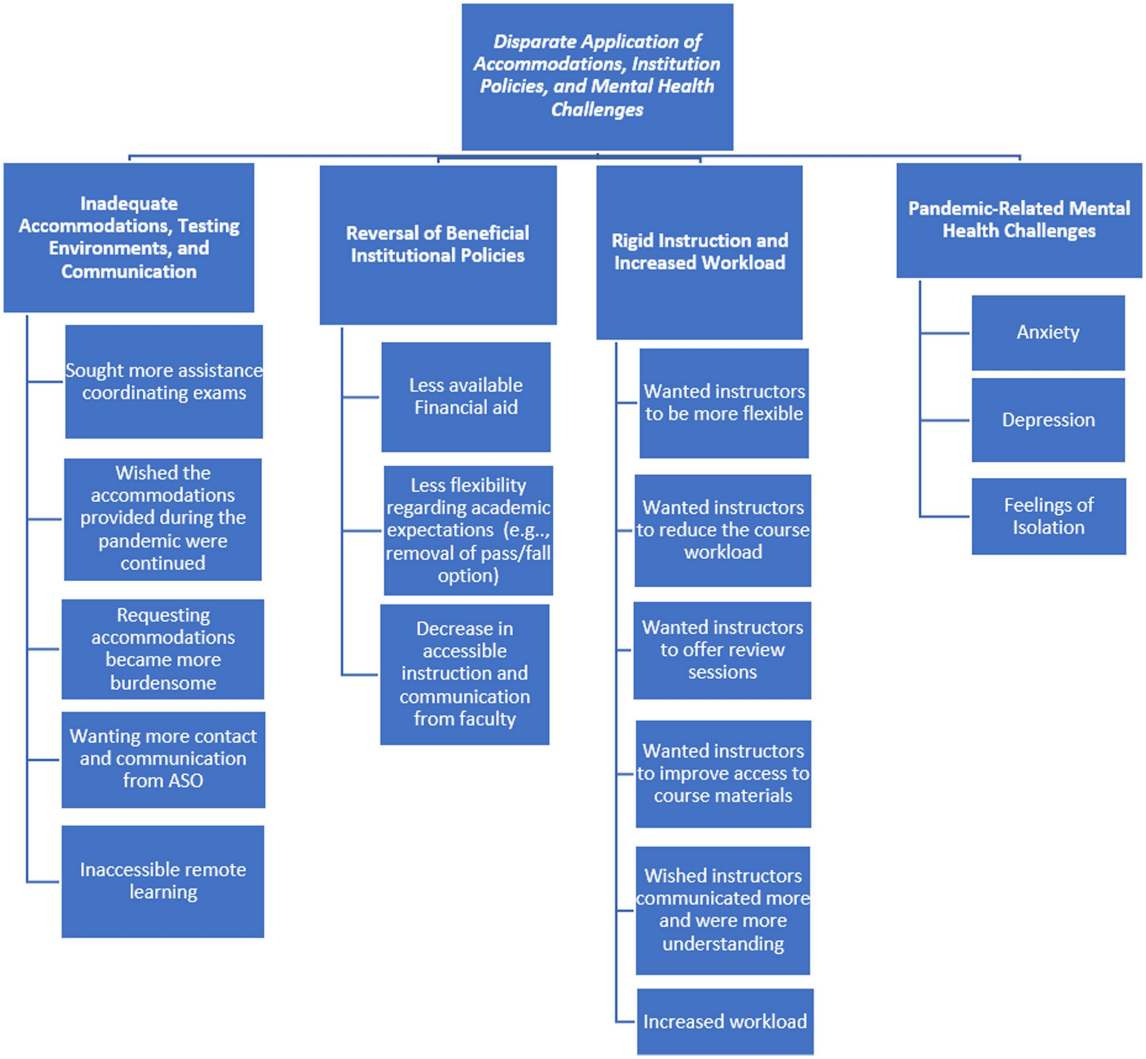
Ongoing pandemic challenges during Fall/Spring 2020–2021 semesters coding tree.

Thematic analysis was used to examine the open-ended responses, which allowed participants to expand upon and clarify their responses to the closed-ended survey questions. First, authors five and six gained familiarity with the data by reading through participant open-ended responses and noting initial patterns in the overall dataset. Each author independently identified and labeled initial codes, or salient aspects of the data that related to the research questions. The authors met to compare codes and resolved any discrepancies. Next, the authors independently collated codes into emerging themes, and again met to discuss themes and resolve discrepancies. The authors reviewed the themes to ensure they represented and encompassed the codes within them, refined theme names and generated a coding map (see Figures 1, 2) to document the analytical process (Braun and Clarke, 2012). Researcher triangulation, as well as including an audit trail *via* the coding map, were carried out to provide transparency of the analysis and to establish trustworthiness of findings.

## Results

### Quantitative Results

As depicted in Table 1, the sample was predominantly Female (*n* = 60) and were enrolled at four-year institutions (*n* = 64). Mental health concerns (*n* = 47), ADHD (*n* = 33), and learning disabilities (*n* = 33) were the most common disability categories reported by the participants. It is important to note that participants could select multiple disability categories and 53 participants did so. Survey respondents were asked to indicate where they lived during the academic year. Thirty-four participants indicated living at home and 31 participants reported living off campus (e.g., in a rented house or apartment). Eighteen participants reported living on campus and four participants spent one semester at home and one semester on campus during the 2020–2021 academic year.

**Table 1:** Demographic characteristics of the sample.

Demographic	*n*	%
**Gender**		
Male	17	19.3%
Female	60	68.2%
Nonbinary	10	11.4%
Prefer not to say	1	1.1%
**Disability N**		
One	35	39.8%
Two or more	53	60.2%
**Disability Type**^1^		
ADHD	33	37.5%
ASD	22	25.0%
Chronic Health	22	25.0%
Deafness/Hard of Hearing	6	6.8%
Mental Health	47	53.4%
Intellectual disability	4	4.5%
Learning disability	33	37.5%
Mobility/Orthopedic disability	10	11.4%
Speech/language impairment	2	2.3%
Traumatic or acquired brain injury	6	6.8%
Visual impairment (including blindness)	7	8.0%
Other	12	13.6%
**Degrees Currently Pursuing**		
Associate’s Degree	6	6.8%
Bachelor’s Degree	64	72.7%
Graduate Degree	16	18.2%
Fall 2020 Graduate	2	2.3%
**Living Situation**		
At my family home	34	38.7%
In a residence hall	18	20.5%
In my own residence	31	35.2%
At home for one semester and on campus for another	4	4.5%
Missing	1	1.1%
Tested positive for COVID-19	7	7.9%
Had to quarantine	30	34.1%

1 *As participants could select more than one response, the sum of the disability categories will add up to more than 88*.

Thirty participants noted having to quarantine at least once during the 2020–2021 academic year and 22 of these participants stated quarantining had a negative impact on their academic experience. The participants were also asked to indicate if they tested positive for COVID-19 during the 2020–2021 school year and seven revealed that they had. Of these seven, six stated the illness had a negative impact on their academic experience.

Participants were asked to indicate the type and format of remote instruction received during the 2020–2021 school year, and they noted that a variety of instructional strategies were implemented. Remote instruction utilized approaches including materials uploaded to a LMS (*n* = 80), instructional e-mails regarding updates and changes (*n* = 80), and video lectures (*n* = 79). As noted in Table 2, participants also stated courses were offered in a variety of formats including online synchronous (*n* = 71), online asynchronous (*n* = 41), and online courses employing both synchronous and asynchronous elements (*n* = 38). In addition, 63 participants reported having more than two course formats across the 2020–2021 school year. Participants were also asked to indicate if they had placed a course on pass/fail and 19 indicated they had done so with at least one course.

**Table 2:** Type and format of remote instruction received by the sample.

Course instruction type/format^1^	*n*	%
**Remote instruction type**		
Use of an instant messaging service	6	6.8%
Video Lectures/Classes	79	89.8%
Materials uploaded to a college LMS	80	90.9%
Emailed instructional documents directly	35	39.8%
Email communication regarding updates, changes, etc.	80	90.9%
In-person	25	28.4%
Other	1	1.1%
**Course Formats**		
*Format*		
At least one course used a combination of synchronous and asynchronous formats	38	43.2%
At least one course was delivered asynchronously	41	46.6%
At least one course was delivered synchronously	71	80.7%
At least one course was conducted completely in-person	20	22.7%
At least one course was conducted as a blend of in-person and remote	25	28.4%
Missing	1	1.1%
**Number of Formats**		
1	25	28.4%
2	29	33.0%
3	22	25.0%
4	9	10.2%
5	2	2.3%
Missing	1	1.1%

1 As participants could select more than one response, the sum of the disability categories will add up to more than 88.

Participants were asked to compare their experiences during spring 2020 to the 2020–2021 academic year by emphasizing differences between spring 2020 and spring 2021 (see Tables 3 and 4). In general, SWD reported their experiences were similar, but demonstrated a generally positive trend (scores >3.00) for institutional-related experiences (Table 4). For example, SWD noted their instructors were more flexible during the 2020–2021 school year and that both the quality and accessibility of remote platforms used to deliver instruction improved. Students also reported learning materials were more accessible and that they received more support and improved communication from their instructors. Alternatively, responses demonstrated a generally negative trend (majority of scores <3.00) regarding personal experiences (Table 3). For example, participants rated their mental health, motivation, connection with peers and financial concerns as worse than in spring 2020. Time management and the ability to be productive in one’s workspace were also rated as worse in the spring 2021 semester.

**Table 3:** Personal experiences comparing Spring 2020 and 2020–2021 School Year Likert Scale Questions.

Item	*n*	Mean	*SD*	*1*	*2*	*3*	*4*	*5*
Mental health concerns as a result of the pandemic (e.g., anxiety, depression)	88	2.23	1.10	27	28	23	6	4
My motivation to learn	88	2.59	1.64	29	24	15	14	6
My connection to other students in my classes	88	2.64	1.63	27	24	17	14	6
Financial concerns as a result of COVID-19 that impacted my college career	88	2.76	0.93	10	17	49	8	4
My ability to manage my time	88	2.77	1.51	18	27	23	14	5
My ability to be productive in my workspace	88	2.77	1.53	16	35	16	16	5
Issues with food insecurity	88	2.85	0.58	4	10	69	5	0
My ability to set and keep a consistent schedule	88	2.87	1.54	15	32	18	18	5
The impact of family demands (e.g., caring for family members) on my learning	88	2.88	1.30	12	21	40	11	4
Medical concerns related to COVID-19	88	2.91	0.81	1	23	54	3	7
My ability to take notes	88	2.93	1.58	16	24	28	10	10
The reliability of internet access in the place that I live	88	3.07	0.83	3	13	52	15	5
My access to a personal computer/laptop that no one else uses	88	3.11	0.67	2	3	72	5	6
The security of my housing situation	88	3.24	1.07	3	9	60	12	4

Scale: 1: Much worse than spring 2020, 2: Worse than spring 2020, 3: No different than spring 2020, 4: Better than spring 2020, 5: Much better than spring 2020.

**Table 4:** University policy-related experiences comparing Spring 2020 and 2020–2021 School Year Likert Scale Questions.

Item	*n*	Mean	*SD*	*1*	*2*	*3*	*4*	*5*
Receiving the accommodations I need in my classes	88	3.01	0.92	7	11	48	18	4
My connection to my instructors at my college/university	88	3.02	1.51	13	24	28	17	6
The support from my accessibility services center	88	3.07	1.18	7	15	50	13	3
My ability to access student supports (e.g., counseling, advising, career services)	88	3.11	1.35	10	15	43	15	5
The support from my institution	88	3.12	1.35	10	13	46	13	6
Receiving clear communication from my instructors	88	3.15	1.03	5	18	32	25	8
The ease of contacting my accessibility services center and receiving a response in a timely manner	88	3.19	1.21	8	7	55	14	4
My access to needed disability-related service	88	3.20	1.22	7	11	50	17	3
The guidance I received related to how to use any new technologies/software needed for my courses	88	3.25	1.28	7	9	53	12	7
The communication from instructors about any changes to assignments and projects	88	3.42	1.37	3	16	45	13	11
The accessibility of the remote platform(s) used to deliver instruction	87	3.48	1.32	4	10	47	18	8
The support from my instructor	88	3.51	1.43	6	14	36	25	7
The accessibility of institutional/learning material	88	3.52	1.36	5	11	42	23	7
The quality of the remote platform(s) used to deliver instruction facilitated learning	87	3.63	1.45	6	10	37	25	9
The flexibility of my instructors related to course design (e.g., course assignments deadlines, etc.)	88	3.77	1.42	4	12	32	32	8

Scale: 1: Much worse than spring 2020, 2: Worse than spring 2020, 3: No different than spring 2020, 4: Better than spring 2020, 5: Much better than spring 2020.

### Open-Ended Responses

Open-ended responses allowed participants to expand upon their quantitative responses regarding experiences in the 2020–2021 academic year. Improvements made by accessibility services offices (ASOs), institutions, and professors can be characterized by the theme: Increased clarity and expansion of institutional procedures, which encompassed three main categories, including (a) expanded/increased clarity and communication from ASOs, (b) institution wide improvements involving increased financial, learning and COVID-related supports and communication, and continuance of flexible policies, and (c) increased faculty communication, availability and accessibility. Participants also discussed ongoing pandemic challenges, which involved (a) retraction of beneficial adaptations to accommodations, accommodation procedures, and institutional policies put in place as the pandemic developed, (b) continued challenges to accessible remote learning, (c) changes made by faculty, including reduced communication, reductions in accessible teaching practices, and increased workload, and (d) pandemic-related mental health challenges. Each area is described below.

#### Improvements Made as Institutional Policy and Practice Adapted to the Pandemic

Participant data indicate that as the pandemic continued, accessibility services professionals, institutional policy makers, and professors continued or expanded beneficial practices used during the initial shift to remote instruction.

##### Expanded/Increased Clarity and Communication

While 22 participants indicated the ASO at their institution did not make any changes, 24 stated the office expanded procedures to include those that had been particularly helpful during the pandemic, including providing increased clarity regarding how to utilize accommodations in remote learning environments, and communicating more frequently with students. One student, when making comparison to previous semesters, stated the office provided “more communication and had detailed explanations of how accommodations would work for remote exams.” In another spring 2020 to spring 2021 comparison, a student described how the ASO was better able to provide in person support. The student said:

During the spring of 2020 I lived at home, so they were unable to support me at all aside from whatever supports might be given for online learning, which accommodations I do not use. This semester, however, I am on campus, which means that they are able to support me by arranging the accommodations that make it safer for me to live on campus, away from home.

Students also noted their ASO communicated more proactively. One said the office “was more attentive and it was easier to both get in touch with them and set up accommodations letters,” while another said their accessibility services office was “helpful in making sure I received frequent updates to the resources at my disposal.” Seventeen participants reported feeling satisfied with the services their campus accessibility services offices provided.

##### Institution-Wide Improvements

Thirty-six participants reported institution-wide improvements, including those related to increased financial support, remote learning support, increased communication, flexible university policies, and improved COVID-19 protocols. Regarding COVID-19 protocols, students were pleased there were increased opportunities to receive virus testing and vaccines, and specific guidance to professors and students regarding how to respond to absences, extensions, and class policies. One student described that the institution:

provided clear expectations about whether classes were in-person or remote, and if remote, synchronous or asynchronous, and they held professors to those expectations – professors could not require in-person exams for an asynchronous remote class. Knowing the expectations rather than having it be different from professor to professor was helpful.

##### Increased Faculty Communication, Availability, and Accessibility

Regarding academic experiences, several students reported their professors communicated more, had more availability, and were better equipped to deal with remote instruction. Twelve participants stated their instructors were more understanding, empathetic, and compassionate. One student described, “these past few semesters seemed to increase understanding and empathy for the students’ possible mental health state in their classes. Most also gave ample extensions and considerations for their students if they requested it.”

Participants also credited instructors with providing virtual class meetings and more accessible classes including clearer deadlines, improved access to course materials, and more flexibility. For example, ten participants stated they were satisfied with the support provided by their instructors in the spring 2021 semester. Some students noted a desire for instructional practices they found beneficial to continue when face-to-face instruction resumes, including increased course accessibility, flexibility, instructor availability, and the use of varied instructional methods, such as breakout rooms, additional remote study sessions, and the use of asynchronous course activities.

#### Ongoing Pandemic Challenges During Fall/Spring 2020–2021 Semesters

While some participants described benefits due to pandemic-driven modifications, others found those beneficial modificaitons withdrawn despite ongoing pandemic challenges. Participants also reported some policies, including accommodation-related policies and procedures, were never effectively adapted to novel remote learning settings, with other policies and procedures becoming even more burdensome. Participants also shared pandemic-related mental health challenges. Each is described in more detail below.

##### Inadequate Accommodations, Testing Environments, and Communication

Eleven participants reported that their ASO could have better adapted their accommodations for remote instruction or better communicated with faculty about exam accommodations. Students often cited the inaccessibility of testing in remote coursework. One student described her challenging experience:

In non-covid semesters, I have taken my tests in the [accessibility services] testing room. This has been a very effective accommodation for me. Once we switched to the online format, the [accessibility services office] stopped providing testing administrators and began requiring students with testing accommodations to just work it out with their professors. Not only did this result in undue burden on professors already coping with restructuring their teaching, but it had a severely negative impact on me. Taking my tests with the other students in my class was very distracting.

The student said the most distracting features of remote testing included announcements about the exam time remaining (which did not apply to this individual, who received extra time to complete the exam), beeps indicating when a student entered or left the remote room, and hearing student conversation as exams were submitted. Another student offered a potential solution to these issues, suggesting that accessibility services offices “should be offering a virtual version of their testing office. In regular semesters the university already pay [*sic*] someone to sit in the testing office so I do not see why the university could not have someone sit in a virtual room and do the same thing.”

Other participants stated they wished the accommodations provided during the pandemic were continued, including the option to forgo in-person instruction. For example, several students thought the process to request accommodations became more burdensome, as they had to notify an accessibility services professional, as well as their instructor. One student described how these new procedures placed increased demands on students’ executive functioning:

Although it could have helped in some cases enforcing accommodations, the logistics of emailing the service provider with the professors is a bit irksome and difficult, requiring the communication between a lot of people on items which could be of short notice. Especially for individuals with executive functioning issues, this makes getting help difficult.

Another student shared how this new process created additional mental health burdens. The student stated, “if I am experiencing a depressive episode, explaining it to my professor and my [accessibility services] advisor, AND following up with them BOTH afterwards is mentally taxing and it creates anxieties that make me want to stay silent.”

Fifteen participants reported that more contact and communication from the accessibility services office would have been beneficial. One student described,

They could have checked in with me to see if there were any new challenges in the online format that should be addressed or that they could help with. I found myself facing new struggles I have never encountered before with the new online format.

Inaccessible remote learning was reported by nine participants who shared that some instructors did not provide universal captioning, recorded lectures, and transcripts. Participants also recounted an interest in more online services, including time management or motivation focused workshops, as well as opportunities for other support, such as strategies for dealing with loneliness.

##### Reversal of Beneficial Institutional Policies

Fifty-four students indicated they were displeased with the reversal of policies put in place during the pandemic, especially those that resulted in additional financial aid and flexibility regarding academic expectations. One student shared, “My institution as a whole actually is providing less support than spring 2021 [2020; *sic*] because they have treated the pandemic as less and less of a crisis as time went on.” This individual cited the removal of pass/fail policies and receiving less financial aid, which were two benefits available as the pandemic unfolded. Students also described discontent with what they perceived as a decrease in accessible instruction and communication from faculty. One student said, “in the beginning of the pandemic the university encouraged everyone to be extremely flexible and understanding, extended university deadlines, changed schedules, etc. – all of these things have decreased in the past year.” Additionally, when asked what things their institution did better to support them in the spring 2021 semester versus the spring 2020 semester, 18 students responded “nothing.”

##### Rigid Instruction and Increased Workload

Other participants felt they could have been better supported academically if instructors were more flexible, reduced the course workload, offered review sessions, and improved access to course materials. Several participants reported they wished instructors communicated more and were more understanding of issues that arose for them. Students described classroom expectations returning to pre-pandemic levels, despite the pandemic continuing. One student stated the workload increased, writing “among me and my peers, it’s been agreed upon that courses got tougher in spring 2021. There were more assignments that took more time and lectures that were longer than they would have been if we were in-person.” The increasing workload was challenging for many students to manage, given that they reported continuing to deal with pandemic-related difficulties. One student shared, “they (faculty) should just be more understanding that we cannot absorb as much information as before,” and that he/she/they wanted professors to reflect on “students’ ability to perform at the moment.”

##### Pandemic-Related Mental Health Challenges

Ten SWD that reported having a tested positive for COVID-19 experienced academic challenges including falling behind and mental health challenges, such as anxiety, depression, and feelings of isolation. Thirty-four students who were required to quarantine described experiencing both isolation and mental health challenges, as well as academic difficulties due to working from home. One student said, “I was unable to go to classes in-person, so I missed out on material. Additionally, I had to go to my house which was an environment that was much harder to focus in, due to the distractions.” Another student described how working from home decreased his/her/their motivation: “I found myself not enjoying school anymore, I had the mindset of just going through the motion to get assignments done.” Other students described how quarantining caused them to think more about the pandemic, which contributed to mental health challenges and negatively affected academics. One student indicated these circumstances “created immense anxiety and fatigue, which lead to me struggling with assignments,” and “the stress of knowing the impact of COVID and being in such a high-risk category made concentrating absolutely impossible.” Another student indicated that “it was hard not to see my friends or extended family. I felt very lonely and unmotivated to do any schoolwork.”

## Discussion

The present study is a follow-up on the research of Madaus et al. (2021) of postsecondary SWD to compare pandemic-related experiences in spring 2020 with experiences in spring 2021. Comparisons of this nature may illustrate the current state of service delivery, as well as uncover evolving needs of SWD as the pandemic continues. Further, analysis of the results illuminates the complex and individualized nature of how university policy and practice differentially impact SWD. Consequently, following up on students’ previous experience affords a reliable anchor point for comparison. Trends in the data are reported in the following sections, along with study limitations, potential future research opportunities, and implications for accessibility services staff service delivery as the pandemic continues and eventually becomes endemic.

### Varied Experiences of SWD

A major goal of the current study was to compare the experiences and perceptions of the same group of SWD 1 year later regarding various practices employed by their institution. For example, 11 of the 14 experiences reported on were below a mean of 3.0, raising genuine concerns about how SWD are responding to the persistent challenges associated with the pandemic. As policy makers, practitioners, and researchers review the pressing challenges experienced by students during the pandemic, it may be especially important keep in mind the following significant issues higher education and disability stakeholders are encouraged to consider.

#### Course Delivery Methods Varied

The rapid response to the virus in spring 2020 resulted in almost all universities embracing digital delivery options to continue academic instruction. Participants in the current analysis indicated remote learning has continued with almost 90% or more of students receiving video lectures/classes (e.g., Zoom, Teams, WebEx), instructional materials uploaded to an LMS, and email communication for subsequent course updates and changes. Emailing instructional documents to students directly (<40%) was less commonly used by faculty, though the practice was still used in some cases. While some LMSs include an instant message (IM) feature for the dissemination class-wide information, only 6.8% of students reported the use of IM, indicating its considerable lag behind the use of email by faculty. Given the popularity of IM for student communication, this may be a constructive but underutilized mechanism for communication.

However, it was clear from the study respondents that course delivery methods utilized by instructors varied widely and lacked the consistency reported by McDaniel et al. (2020). While a breadth of options is not particularly notable given the individualized nature of institutional responses, what is concerning is that almost 15% of students simultaneously experienced at least four different types of instructional formats. For SWDs in particular, the challenge of navigating multiple instructional delivery formats adds a novel layer of complexity that likely consumes finite executive functioning resources, thus having the potential to negatively impact academic outcomes. Unfortunately, this finding is consistent with the challenges reported by Madaus et al. (2021) regarding the initial spring 2020 shift to virtual learning as SWD struggled with multiple course formats and methods of communication, and it points to the benefit of institutions streamlining these approaches to the greatest extent possible.

#### Decentralized Accommodations

Students emphasized the decentralized nature of accommodations, or leaving the implementation of accommodations to the course professor without sufficient administrative or university support, led to some students experiencing an erosion of approved accommodations similar to the obstacles reported in McDaniel et al. (2020). For example, the experience of the student forced to take exams with other students in a distracting online setting is potentially an indication that the professor may not have fully understood “separate exam location” applies to online examinations as well. Considered through the lens of the principle known as Occam’s Razor, without university capacity, faculty forced to find their own solutions will typically use the simplest solution. In this circumstance, faculty chose to offer the exam remotely to all students identically, with the likely belief by faculty that a “separate exam location” was being honored because the student was completing the exam alone in a remote setting. Such an interpretation, while possibly unintentional, is antithetical to meaningful equal educational access for SWD and reinforces concerns about distractions in the home environment noted by both Madaus et al. (2021) and Valenzuela (2020).

Additionally, a related challenge was that in many cases the new learning conditions required individuals to seek out new accommodations. As one student noted about faculty communication, the online environment presented new and unanticipated challenges. Without proactive faculty instructional design (e.g., Universally Designed Instruction), or communication probes to students about learning challenges, some students found themselves back at the beginning, needing to identify learning challenges and advocate with accessibility services staff to receive new accommodations.

#### Self-Advocacy and Access to Accommodations

Study findings echo the concerns in the field regarding the particular challenges faced by SWD during the pandemic (e.g., McDaniel et al., 2020; Aquino and Scott, 2021; Rodriguez et al., 2021). For example, it is not uncommon for students with high incidence disabilities (like those commonly participating in higher education) to struggle with executive functioning which was exacerbated by pandemic conditions. Whether as a result of a learning disability, ADHD or other disability, asking students to self-advocate, coordinate their own services across multiple professors and learning platforms, and organize assessment conditions places a high burden on those students identified as eligible for accommodations intended to reduce the burden of such executive functioning tasks.

Further, on or off campus residential status likely impacts the way students utilize campus services and has the potential to differentially effect those unable to reliably access institutional resources. Moreover, those 35% of students reporting “independent living” or 39% living at “home” are not necessarily residing within proximity to the university like those in campus residential facilities, resulting in the potential for more significant challenges when a need arises for students to access campus services.

#### Shifting Impact of University Policy

Survey responses echo several key positives related to university policy and practice. For example, almost 30% of respondents commented on accessibility services expanded procedures regarding application for services, communication, and support for SWD in the fall semester. Additionally, more than 40% of students noted institution-wide improvements including increased financial support, remote learning support, increased communication, flexible university policies and improved virus protocols. Similar to findings from Soria et al. (2020), increased financial support and flexible university policies were and remain critical for alleviating student stress, especially as the pandemic decimated jobs throughout the United States and college students with children had to scramble at times to secure coverage for school-age children and/or childcare.

However, more than 60% of respondents indicated frustration with the erosion of some of the afore mentioned flexible spring 2020 policies as the pandemic entered its second year. Of note were shifts in financial support and university approved flexible academic policies such as pass/fail course options. While many had hoped the 2020–2021 academic year would reflect a return to normalcy, this potential reality never materialized. Consequently, as economic and health-related stress continued to intensify for SWD, university removal of such policies may well have been premature and, as noted, resulted in a negation of conditions designed to achieve equal educational access.

#### Shifting Impact of Classroom Policy

The lack of flexibility during the second year of the pandemic seems to have extended to general course expectations as well, with some students noting professors communicated less and set more fixed deadlines. Again, such changes appear premature given the unpredictable nature of the ongoing pandemic and may reflect that some professors felt the need to or were given instructions by their respective institutions at the time, to shift back to pre-pandemic practices.

Yet this was not the experience of all students. In fact, some students noted faculty increased their level of communication, availability for meeting either virtually or face-to-face, and were better prepared to deliver and support remote instruction. Additionally, students described that some faculty provided flexible ways to participate in class, turn in assignments, and access course materials, which can all be helpful strategies for alleviating student stress while maintaining academic learning expectations. While clearly not universal, it is worth noting there was a differential student experience, and therefore likely an array of faculty beliefs for implementing potentially barrier reducing practices.

#### Prolonged Impact on Mental Health

Clearly, COVID-19 had a direct impact on both the physical and mental health of SWD who either contracted the virus or experienced quarantine restrictions. Survey respondents in this study reported a prolonged impact and this mirrors trends reported by Lederer et al. (2021). In particular, the negative impact on 22 students reporting being quarantined is of potentially great concern to accessibility services staff as it raises a critical question: Why was learning negatively impacted if individuals were only quarantined but did not contract the virus? Consider the case of a health condition; we suggest student affairs, accessibility services staff and faculty contemplate additional collaboration regarding how to support students with health conditions that impact learning (e.g., how is makeup learning and testing managed? Are there accommodations or supports that may allow the student to be more successful in the future?). Alternatively, for students quarantined but having not contracted COVID-19, negative learning experiences may become a disincentive to disclose the illness to the university. Such circumstances could result in a larger pool of potentially contagious students engaging in campus activities and therefore increasing overall university community exposure in direct contrast to the purpose of quarantine policies.

### Implications

Lessons learned by comparing spring 2020 to spring 2021 provide an important lens through which colleges and universities can evaluate accessibility throughout their instructional and service delivery system. As virus conditions persisted across the 2020–2021 academic year, academic and co-curricular challenges that marginalized SWD and prevented equitable participation were the result. Reflection regarding mental health, classroom experiences, level of communication, accommodation match, and technology supports are recommended.

#### Student Mental Health

It was predicted the COVID-19 pandemic could persist (Messina et al., 2021) and continue to impact students’ curricular, co-curricular, and personal experiences (Madaus et al., 2021). This unfortunate prediction has, indeed, come to pass. In fact, as we prepare this manuscript many colleges and universities have again opted to shift to fully remote course delivery due to the Omicron variant of COVID-19 (Moody, 2022). Students are unsurprisingly reporting “COVID fatigue” with accounts that more than 90% of all college students have indicated they are experiencing negative mental health symptoms as a function of the pandemic (Dennon, 2021). Current study results revealed that student social–emotional wellness has decreased since spring 2020, which is an indication of persistent pandemic driven mental health challenges. This highlights the importance of two recommendations shared by Madaus et al. (2021) as the pandemic initially impacted colleges and universities, which noted institutions consider: (1) providing epidemiological training, knowledge about COVID-19 and transmission prevention measures, with particular consideration of unique populations such as disabled students, and (2) the rapid provision of mental health services for students and training for campus personnel to spot signs of social–emotional distress in students.

#### Delivery of Instruction

Based on the results indicating an increasing role in accommodations, faculty would benefit from professional development in inclusive pedagogy that could emphasize, for example, that equitable is not always equal. Additionally, training in the value of universally designed instruction would emphasize the use of regular recording of class lectures, providing closed captioning for all recorded viewing, and posting of all course materials to the institution’s LMS. Regular communication should be a pillar of academic practice and is frequently already in place. For example, the desire for deadline flexibility as shared by students in this follow-up study is already a part of the student affairs notification when students have any health-related or athletic reason for a class meeting absence. Faculty might benefit from guidance regarding when to consider and how to assess a need for the use of flexible deadlines for all students. Classrooms that embrace the inclusive practices described are likely to be more welcoming, lead to improved student learning, improved faculty assessment ratings, and could be used by universities to measure campus wide inclusive efforts.

In addition, now that remote delivery of academic coursework has been developed and delivered across a period of years, it is fair to surmise it is an instructional method that will continue to be utilized. Institutions may also be considering or even currently utilizing modified approaches to fully online learning, such as the HyFlex model (described in more detail below; Messina et al., 2021), thus increased student participation in online coursework should be expected by accessibility services professionals both now and into the future.

Respondents in this study, and others (Goldrick-Rab et al., 2020; Means and Neisler, 2020) present with a wide range of academic and social emotional needs suggesting institutions consider utilizing instructional models that better support a diverse range of students. Colleges and universities (e.g., University of Florida, University of San Francisco) have begun to implement an instructional delivery model that allows for a fluid transition between face-to-face and online course participation known as the HyFlex learning model. This model utilizes hybrid instructional techniques with additional flexibility for course participation (Beatty, 2014). Students in HyFlex courses have the option of attending synchronously online or face-to-face, and can alter their choice each course meeting or even based upon course subject matter. That is, students make decisions about content delivery mode based upon their preference and needs (Malczyk, 2019). In such a scenario, course instructors are responsible for providing an equivalent experience for students whether in person or online (Beatty, 2010). As the pandemic experience has highlighted, HyFlex models can be particularly efficacious in circumstances in which systemic (e.g., inclement weather) or personal circumstances (e.g., childcare needs) result in a need to deliver or participate in coursework in a range of formats.

### Communication With Students

Communication can exist in a variety of forms. In the past, faculty relied on opportunities during face-to-face instruction to address student queries. However, the pandemic has frequently limited these interactions for a variety of reasons, leaving students often feeling isolated and outside their typical learning setting. As a result, faculty are encouraged to consider consistent communication outside of the class setting using the institution’s LMS for email, IM, and other ways of communicating information. Updates might include assignment deadlines or clarifications, confirmation of class meetings and modalities (synchronous or asynchronous), or just a regular status check-in to encourage to student communication. Finally, faculty availability before and after class, and availability during regularly scheduled office hours (in both cases, in-person or remote) are indicators of willingness to meet student needs and promote general course accessibility and student success.

In the current study, remote course delivery and remote access to campus services, interestingly, resulted in several accessibility improvements according to student participants. Students reported that instructional and institutional flexibility with respect to course participation and interaction with campus services, including accessibility services, are worthy of continuation. For example, students found that building in flexibility in participation modes and assignment deadlines improved their course experience. Additionally, they reported flexibility with respect to mode of participation in campus meetings also allowed for an improvement in their college experience. Respondents suggested campus meetings, workshops, and courses continue to be offered remotely. In fact, the suggestions students described are common characteristics of practices naturally built into courses designed and delivered using UDI principles (Faggella-Luby et al., 2019).

Regardless of mode of course delivery, students in the current study reported several curricular and co-curricular pandemic driven experiences they would like continued in the future. First, institutions should encourage all personnel to evaluate the nature and consistency of their communication with students. Respondents noted they benefited from personnel consistently communicating with them, especially when that interaction expressed empathy and understanding of their circumstances. Some students described the value of remote delivery of both wellness and student success workshops. Specifically, they noted an interest in the continued online delivery of functions addressing skills such as time management, and notetaking. Next, they reported a desire to have the ability to access support services, participate in small study groups, and engage peers informally in online environments. Lastly, and the current authors have received requests of this type, students are requesting faculty be trained in both improving course accessibility and the effective use of LMSs.

### Delivery of Accommodations

Typically, students review the identification and provision of accommodations each semester and do so at the course level. However, given the large numbers of students in this study participating in an array of instructional modalities, across a multitude of disciplines and varying levels of instructor inclusivity practice, perhaps policy should reflect the need to regularly review accommodations at the level of course modality as well. For example, exam accommodations might look differently during in-person testing in comparison to remote settings. Accommodation match is critically important to meeting institutional accessibility services legal mandates, academic program accessibility and student success.

### Technology Supports

University technology staff, instructional development staff and instructors are encouraged to keep in mind the benefits to all learners of an effective and efficiently implemented LMS. This includes platforms for posting class content (e.g., Canvas), but also for communication (e.g., email, Zoom). While some platforms frequently come at great expense, their potential for providing accommodations such as closed captioning, storing class recordings and posting of other relevant materials are invaluable to *all* students. Self-reflection might include consideration of how use of these tools is evaluated, by whom and potential barriers for student, and faculty use.

### Opportunity for Student Reflection

Accessibility services personnel could also use the opportunity for self-reflection to encourage and support SWD consideration of academic learning strategies, executive functioning, and overall appropriateness of approved accommodations. Students are obliged to self-advocate in the future when seeking employment, and an ideal opportunity to practice this important skill is while one is in college utilizing supports under the guidance of accessibility services professionals. Such practice is fundamental to self-determination and a critical life-long skill for SWD. Moreover, by participating in the process, accessibility services personnel will better be able to assess institutional efficacy regarding SWD learning support.

### Limitations

While the current study builds upon previous research, the study outcomes should be interpreted with caution. First, the study is made up of student self-reported data and the report of both their disability and campus status and academic experience has not been verified in any manner. Secondly, though the initial study from which this sample was drawn utilized several networks and national organizations to gather respondents, a considerable majority of the sample reported mental health, ADHD, and learning disabilities, with other disabilities being reported far less frequently. Additionally, respondents were also mostly female. Lastly, the original survey, which was used as the basis for the current study, was developed utilizing two validated measures, however, it was in the end a unique questionnaire and was not pilot tested.

Finally, it is important to note that the nature of the question asked requires students to compare two points in time, not one set of experiences to a benchmark of quality, potentially causing a regression to the mean. In other words, it is possible that students had an inflated sense of how they were thriving during the early stages of the pandemic (spring 2020) and are now reevaluating their status in a more negative light (spring 2021). Similarly, students may have been frustrated with their institution’s rapid response in 2020, and little to no differences in policy and practice might appear in the spring 2021 data as “no difference.” Therefore, it was intentional to review the qualitative open-ended responses for further explanation and to highlight student voice in the process.

### Summary

The purpose of the current study was to examine the pandemic-related SWD experiences and perceptions of the 2020–2021 academic year. It was designed to build upon a previous investigation (Madaus et al., 2021) conducted shortly after the pandemic began; therefore, the sample of the current study was culled from the analysis by Madaus and colleagues. Comparisons across time, utilizing the same respondent pool, have the potential to reveal ongoing and developing needs of students. The current sample included 88 respondents that reported their motivation, mental health, and perceived connection with their peers have all gotten worse over time. Regarding communication and support of both campus faculty and accessibility services some students, notably, reported improvements across time. In sum, SWD report both ongoing challenges as well as some benefits since the onset of the pandemic in spring 2020. Ultimately, the study results illuminate the complex and individualized nature of the student experience during this anomalous stretch of time.

## Data Availability Statement

The raw data supporting the conclusions of this article will be made available by the authors, without undue reservation.

## Ethics Statement

The studies involving human participants were reviewed and approved by University of Connecticut Institutional Review Board. Written informed consent for participation was not required for this study in accordance with the national legislation and the institutional requirements.

## Author Contributions

JM, MF-L, LD, and NG developed and administered the survey. NG conducted the quantitative analysis. ET and SL conducted the qualitative analysis. JM, MF-L, LD, NG, SL, ET, and AT were involved in writing and editing the manuscript. All authors contributed to the article and approved the submitted version.

## Conflict of Interest

The authors declare that the research was conducted in the absence of any commercial or financial relationships that could be construed as a potential conflict of interest.

## Publisher’s Note

All claims expressed in this article are solely those of the authors and do not necessarily represent those of their affiliated organizations, or those of the publisher, the editors and the reviewers. Any product that may be evaluated in this article, or claim that may be made by its manufacturer, is not guaranteed or endorsed by the publisher.
